# Effects of Omega-3 Fatty Acids on Immune Cells

**DOI:** 10.3390/ijms20205028

**Published:** 2019-10-11

**Authors:** Saray Gutiérrez, Sara L Svahn, Maria E Johansson

**Affiliations:** Department of Physiology, Institute of Neuroscience and Physiology, The Sahlgrenska Academy, University of Gothenburg, 405 30 Gothenburg, Sweden; saray.gutierrez@gu.se (S.G.); sara.svahn@gu.se (S.L.S.)

**Keywords:** polyunsaturated fatty acids, PUFAs, omega-3 fatty acids, α-linolenic acid, ALA, eicosapentaenoic acid, EPA, docosahexaenoic acid, DHA, immune cells, immune response, phagocytosis, immune-modulation, anti-inflammatory, migration, presentation, cytokines, antibody production

## Abstract

Alterations on the immune system caused by omega-3 fatty acids have been described for 30 years. This family of polyunsaturated fatty acids exerts major alterations on the activation of cells from both the innate and the adaptive immune system, although the mechanisms for such regulation are diverse. First, as a constitutive part of the cellular membrane, omega-3 fatty acids can regulate cellular membrane properties, such as membrane fluidity or complex assembly in lipid rafts. In recent years, however, a new role for omega-3 fatty acids and their derivatives as signaling molecules has emerged. In this review, we describe the latest findings describing the effects of omega-3 fatty acids on different cells from the immune system and their possible molecular mechanisms.

## 1. Introduction

The immune system is a defense system that protects organisms from invading pathogens, such as viruses or bacteria. It comprises a heterogeneous group of cells, i.e., immune cells, as well as cell-independent mechanisms. Immune cells can be broadly divided into two main categories according to their properties and defense mechanisms: cells of the innate and cells of the adaptive immune system. Cells from the innate immune system, namely macrophages, neutrophils, eosinophils, basophils, mast cells, natural killer cells, and dendritic cells, are the first cellular line of defense. Their mode of action is generally fast but with limited specificity. Cells from the adaptive immune system, namely B cells and T cells, have a higher level of specificity, but their activation is delayed. However, cells from the adaptive immune system develop memory against pathogens after a first confrontation, and their speed and efficiency against a previously faced pathogen is greatly enhanced during a second encounter.

Coordination of the different immune cells and regulation of their activity is of crucial importance for mounting an effective immune defense. This task is often accomplished by the secretion of cytokines and chemokines, i.e., molecules secreted by cells, including but not restricted to immune cells, that attract immune cells into the site of infection and regulate their activation or their suppression [1,2].

A healthy and balanced diet is essential for the correct function of every part of our organism, including the immune system. Additionally, some dietary factors have been found to have immune-regulatory properties, including micronutrients such as Vitamin D or macronutrients such as fatty acids [3]. The impact of dietary polyunsaturated fatty acids (PUFAs) on the immune system has been investigated for decades, with special focus on the omega-3 PUFAs α-linolenic acid (ALA), eicosapentaenoic acid (EPA), and docosahexaenoic acid (DHA). ALA is found in nuts and seeds whereas EPA and DHA are the main components of fish oil [4,5]. For a comprehensive review of the sources of omega-3 fatty acids, we recommend Cholewski et al. [6].

EPA and DHA can also be synthesized from ALA [7], a process that involves several steps orchestrated by multiple elongases, desaturases, and β-oxidases [8]. However, the synthesis of EPA from ALA occurs at a low rate in mammals [9]. Of note, the same enzymes are employed by omega-6 fatty acids for their metabolic pathways. 

**Figure 1:**
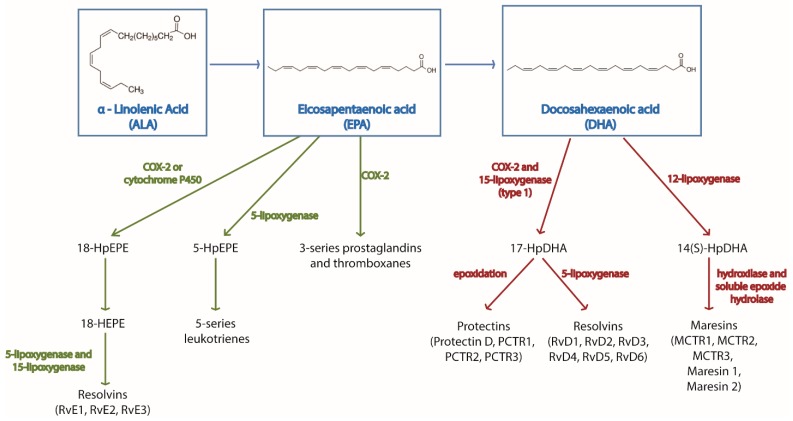
Main biochemical pathways for the synthesis of omega-3 derived metabolites. The figure shows the omega-3 fatty acids α-linolenic acid (ALA), eicosapentaenoic acid (EPA), and docosahexaenoic acid (DHA), their downstream metabolites and the enzymes regulating their synthesis.

Both omega-3 and omega-6-derived metabolites have important immune-regulatory functions. These metabolites are generally known as pro-resolving mediators (SPMs) and can be classified in different families—prostaglandins, leukotrienes, thromboxanes, maresins, protectins, and resolvins. Their synthesis is orchestrated by cyclooxygenase, lipoxygenase, or cytochrome P450 enzymes [10]. A summary of the metabolites produced from omega-3 fatty acids and the enzymes regulating their synthesis is found in Figure 1. Omega-3 and omega-6 substrates compete for these enzymes [11], as well as for the above mentioned elongases and elastases. Therefore, in the presence of omega-3 fatty acids, the competition for the enzymes reduces the synthesis of omega-6-derived metabolites, which also have effects on immune cells. This competition constitutes an additional level of immune-regulation by omega-3 fatty acids.

Although the specific mechanisms of action of omega-3 fatty acid regulation of immune cells function present several cell type-specific features, it is worth mentioning that omega-3 fatty acids, via in vitro stimulation or via dietary supplementation, effectively incorporate into the cellular membrane of all the immune cells investigated to date [12,13,14]. Polyunsaturated fatty acids possess multiple double bonds in their carbon chain. Since each double bond causes a bend in the carbon chain, polyunsaturated fatty acids cannot stack as tightly within cellular membranes as saturated fatty acids do. Therefore, the incorporation of polyunsaturated fatty acids increases the fluidity of cellular membranes [15].

Several outstanding reviews describing the effects of omega-3 fatty acids on the immune system and their clinical effect on immune-related diseases are already available for the reader [16,17,18,19]. The main aim of our review is to describe the specific effects of omega-3 fatty acids on the different types of immune cells focusing on the recent findings on the field (summarized in Table 1).

## 2. Effects of Omega-3 Fatty Acids on Macrophage Function

Macrophages have a fundamental role as part of the innate immune system. They patrol multiple organs in a constant search for invading pathogens. They are able to recognize specific pathogen-associated molecular patterns (PAMPs) thanks to the toll-like receptors (TLRs) present on their surface. After pathogen recognition, they initiate the elimination process of the pathogen by engulfing it (phagocytosis) and secreting anti-microbial molecules such as reactive oxygen species (ROS). Simultaneously, they produce and secrete a large variety of cytokines and chemokines in order to recruit and activate other immune cells from both the innate and the adaptive immune system to mount an efficient immune response to completely eliminate the threat. Macrophages activated by PAMPs and IFN-γ, known as classically activated or M1 macrophages, secrete TNF-α and IL-1β and direct their actions toward pathogen elimination. They produce antigenic peptides from the destroyed pathogen to present them to adaptive immune cells, which subsequently initiate a more specific immune response [20]. In contrast, macrophages can also be activated by IL-4 into alternatively activated, or M2, macrophages, which secrete IL-10 and promote tissue repair [21].

The impact of omega-3 fatty acids on macrophage function has extensively been investigated since the 1980s [22,23]. Since then, there are three main properties of macrophage biology that have been identified to be altered by omega-3 fatty acids: the production and secretion of cytokines and chemokines, the capacity of phagocytosis, and the polarization into classically activated or alternatively activated macrophages. These investigations have been carried out both using in vitro and in vivo experimental approaches. Since the literature on the topic is extremely extensive, we focus in this review on the direct effects of fatty acids on macrophages found in vitro. In such experiments, the alterations observed in macrophage biology are more directly related to the treatment with omega-3 fatty acids and not by the communication with other cell types that could also be affected by the omega-3 fatty acids as it happens in the in vivo investigations.

Omega-3 fatty acids provoke major alterations on gene regulation on macrophages. Treatment of macrophages with DHA or EPA induces major changes in gene expression in THP-1-derived, LPS-activated macrophages [24], although the effects of DHA and EPA are not identical. Whereas most of EPA-regulated genes belong to the cell cycle regulation family, most of DHA-regulated genes belong to immune response pathways. Similarly, the addition of the omega-3 fatty acids DHA and EPA into the growth cell culture medium for macrophages induces significant global changes in miRNA expression profile. Roessler et al. found that the incubation of Raw macrophage cell line with DHA, in combination with or without LPS, results in alterations in the expression of miRNAs regulating pathways related to gene expression, signal transduction, immune defense, growth/differentiation, and transport-associated genes [25].

### 2.1. Regulation of the Production and Secretion of Cytokines in Macrophages by Omega-3 Fatty Acids

Probably the most renowned property of omega-3 fatty acids is their ability to reduce inflammation and their beneficial effect on inflammation-related disorders (we suggest the review on the topic by Calder, 2011, [26]). For some of these diseases, it has been suggested that the cell type responsible for the inflammatory modulation by omega-3 fatty acids is macrophages [27]. To date, the effects of omega-3 fatty acids on the production and secretion of cytokines and chemokines by macrophages have extensively been investigated, as well as the regulatory mechanisms behind them. Mildenberger et al. [28] quantified the levels of 579 inflammation-related mRNA transcripts in LPS-stimulated human primary monocyte-derived macrophages (MDMs) pre-treated with DHA. Twenty-five percent of the quantified transcripts upregulated by LPS were attenuated by DHA pre-treatment. Downregulation of LPS-induced increase in cytokine gene expression by DHA or EPA has also been demonstrated in macrophage cell lines, such as Raw macrophages or THP-1-derived macrophages, as well as in primary mouse macrophages (bone marrow–derived macrophages and primary peritoneal macrophages) [24,29,30,31,32,33,34]. Interestingly, the timing for the addition of the omega-3 fatty acids seems to play an important role. For example, addition of DHA to BMDMs after LPS stimulation does not alter mRNA levels of *TNFα* but it does reduce mRNA levels of *pro-IL1-β* [31]. Allam-Ndoul et al. demonstrated that the most effective downregulation of LPS-induced mRNA expression of *il6*, *tnfα*, *il1β*, and *mcp1* genes in THP-1-derived macrophages was achieved after LPS stimulation for EPA but during LPS stimulation for DHA [35]. Most studies currently employ pre-incubation with omega-3 fatty acids, however, additive effects of co-incubating macrophages with EPA and DHA has been demonstrated in Raw macrophages [36] and in THP-1-derived macrophages [24]. Of interest, pre-incubation of Raw macrophages with DHA also prevents the increase in the mRNA expression of *mcp1*, *il1a*, *il1b*, and *il6* when the inflammation is caused by a combination of LPS and the saturated fatty acid palmitic acid, suggesting that omega-3 fatty acids can effectively counteract the pro-inflammatory effects caused by saturated fatty acids [32]. Last, not only DHA and EPA have been found to decrease gene expression of cytokines in macrophages in vitro, but also other derivatives from linolenic acid [33]. Most of the above-mentioned studies found no changes induced by the incubation with omega-3 fatty acids in macrophages unless stimulated with LPS.

Investigations of the secreted levels of cytokines and chemokines confirmed the above described effects of omega-3 fatty acids on cytokine/chemokine gene expression in macrophages [24,37,38]. Importantly, the anti-inflammatory properties of EPA and DHA on macrophages are not exclusive to LPS-induced inflammation. THP-1-derived macrophages incubated with ox-LDL (to mimic the formation of foam cells seen in diseases such as atherosclerosis) secreted higher levels of IL-6 and TNF-α than control macrophages, an increase that was successfully rescued by the incubation with omega-3 fatty acids [39]. DHA and EPA also decrease cytokine secretion in Raw macrophages that were infected with *R. equi* or *P.aeruginosa* [40] or stimulated with palmitic acid in combination with LPS [32]. Two interesting facts can be drawn as well from these last two studies. First, the anti-inflammatory effect of DHA was more potent than the effect of EPA. Second, the only cytokine whose secretion was increased by the treatment with omega-3 fatty acids was the anti-inflammatory cytokine IL-10.

The omega-3 fatty acids EPA and DHA are able to suppress inflammasome-mediated inflammation with potency similar of that of the classical caspase-1 inhibitor YVAD [41]. The inhibition of the NLRP3 inflammasome by EPA and DHA has been demonstrated in macrophage cell lines as well as in primary human and mouse macrophages [33,41,42] and for wide range of inflammasome-activating stimuli [31,41]. However, the pre-treatment of macrophages with EPA or DHA does not inhibit all kinds of inflammasome. As demonstrated by Yan et al. [31], DHA, EPA, and ALA decrease IL-1β secretion in BMDMs stimulated with LPS and anthrax lethal toxin (activator of the NLRP1 inflammasome) but not in BMDMs stimulated with LPS and infected with *S. Typhimurium* or in BMDMs treated with LPS and poly (dA:dT) to activate the NLRC4 or the AIM2 inflammasomes respectively. A plausible mechanism behind omega-3 fatty acids-mediated inhibition of the NLRP3 inflammasome can be the decrease in the expression of the *nlrp3* gene [33,42]. Interestingly, omega-3 fatty acid inhibition of the NLPR3 inflammasome requires PPARγ and GPR120/GRP40 signaling [31,33].

Inflammation is a tightly regulated process in which many different receptors, signaling pathways, and transcription factors are involved. LPS-induced inflammation is triggered by the direct contact between LPS and its receptor TLR4. The signaling cascade downstream of TLR4 leads to the phosphorylation and nuclear translocation of transcription factors regulating the expression of cytokines, such as NFkB, MAPK, or ERK [43]. Pre-treatment of LPS-stimulated Raw macrophages with DHA, not EPA or ALA, decrease *tlr4* mRNA [44] however, no changes have been found in the gene expression for other factors involved in the TLR4 downstream signaling [44]. Pre-treatment of Raw macrophages with EPA or DHA does not alter the global surface expression of TLR1, TLR2, TLR4, TLR6, or CD14 [38,45], although an increased localization of TLR4 and CD14 upon EPA or DHA treatment has been found specifically in the macrophage membrane lipid rafts [44].

Decreased LPS-induced phosphorylation, nuclear translocation, and transcriptional activity of NFkB by pre-treatment with omega-3 fatty acids have been demonstrated in primary macrophages and macrophage cell lines with EPA, DHA, or the plant-derived omega-3 fatty acid steridonic acid [32,33,46,47]. EPA and DHA are also able to decrease NFkB activation induced by the TLR2 ligand PamCAG [46]. Decreased activation by EPA or DHA treatment has also been described for other transcription factors such as IRF3, STAT-1, STAT-3, IRF1, ERK1/2, JNK, and MAPK [28,47] but not for others such as CREB [34]. However, how omega-3 fatty acids exert this modulation on inflammatory transcription factors activation remains largely unknown. It has been shown that DHA and other ALA derivatives decrease ROS and NO production [33,47] in LPS-stimulated macrophages, which could be associated with decreased transcription factor activation. Also, DHA and EPA have been found to regulate miRNA expression in Raw and THP-1-derived macrophages that could add an additional regulatory level of the inflammatory transcription factors [25,48].

### 2.2. Effects on Macrophage Polarization by Omega-3 Fatty Acids

The evidence supporting the anti-inflammatory properties of omega-3 fatty acids on macrophages, mainly decreasing the secretion of IL-1β, TNF-α, and IL-6, already backs the notion that omega-3 fatty acids blunt M1 macrophage polarization upon LPS stimulation. Additionally, omega-3 fatty acids have been found to promote M2 polarization in macrophage cell lines and primary mouse macrophages [29,30,49]. Treatment of macrophages with ALA, DHA, or other ALA-omega-3 derivatives increases mRNA expression of *Arg1* [49], *cd36*, *il10*, and *tgfβ* [29] and the surface expression of CD206 [49]. These effects were abolished when GPR40 was blocked [49] or PPARγ was silenced [29]. Omega-3-induced polarization of macrophages towards an M2 phenotype has been successfully employed to reduce brain injury upon stroke [50] and alleviate atopic dermatitis [51].

### 2.3. Effects of Omega-3 Fatty Acids on the Phagocytic Capacity of Macrophages

Although the treatment with omega-3 fatty acids decreases M1 polarization of macrophages, ALA, DHA and EPA can increase the phagocytic capacity of macrophages. This omega-3 fatty acid-mediated increase in the phagocytic capacity has been demonstrated for the engulfment of zymosan particles [29], *R.Equi*, *P.aeruginosa* [52], *E.coli* [53], as well as phagocytosis of apoptotic cells [29]. Some investigators have suggested that this increase in the phagocytic capacity of macrophages upon omega-3 treatment could be related to changes in the cellular membrane composition and structure caused by the incorporation of the omega-3 fatty acids [44,54], although causation still remains to be confirmed.

## 3. Effects of Omega-3 Fatty Acids on Neutrophil Function

The most abundant leukocyte in humans is the neutrophil. The vast majority of mature neutrophils are found in the circulation, blood, and the marginal pools (bone marrow, spleen, and liver), whereas a small portion is found in tissues [55,56,57]. Neutrophils are the first cells to be recruited to the site of inflammation [58] and have an important role in the clearance of pathogens. However, neutrophils can also interact with the adaptive immune system by promoting naïve T cells to transition into T helper 1 cells and can present antigens to B-cells in the spleen [59].

Omega-3 fatty acids have been shown to be incorporated into phospholipids in the cell membrane of neutrophils at the expense of the omega-6 fatty acids linoleic and arachidonic acid [13,60]. Once the omega-3 fatty acids have been incorporated into the phospholipids, they can be metabolized by neutrophils into prostaglandins, leukotrienes, thromboxanes, maresins, protectins, and resolvins [10,61,62]. Omega-3 fatty acids, and their metabolites, modulate neutrophil function in several ways, including neutrophil migration, phagocytic capacity, as well as the production of reactive oxygen species and cytokines.

### 3.1. Effects on Neutrophil Migration and Transmigration by Omega-3 Fatty Acids

Several omega-3-derived metabolites have been found to inhibit migration of neutrophils both in vitro and in vivo. The omega-3 fatty acid metabolite resolving D1 reduces neutrophil migration in a microfluidic system [63]. In line with these findings, Krishnamoorthy et al. showed that human polymorphonuclear leukocytes have, after incubation with resolvin D1, a decrease in actin polymerization, which subsequently leads to reduced neutrophil migration [64].

Endothelial cells are involved in the migration of neutrophils by producing prostaglandin D2 (a metabolite derived from the omega-6 fatty acid arachidonic acid). When prostaglandin D2 binds to its receptor, DP-1 on neutrophils, it leads to neutrophil adhesion and transmigration. However, if endothelial cells are treated with EPA, the omega-3 fatty acid induces a switch in the endothelial cells toward the production of prostaglandin D3, with opposing effects compared with prostaglandin D2 [65]. Further, one of the metabolites from DHA, resolvin D3, reduces the number of transmigrated neutrophils in a mice-peritonitis model [66]. Another group of omega-3 fatty acid derived lipid mediators, the maresins, also inhibit neutrophil infiltration [67,68]. Both protectin D1 and aspirin-triggered protectin D1 (AT-PD1) reduce neutrophil infiltration into the peritoneum of mice upon TNF-α-induced peritonitis [69].

Surprisingly, when human volunteers were given fish oil supplementation and blood neutrophil migration capacity was investigated, fish oil supplementation led to increased migration capacity of neutrophils [70]. However, it should be noted that the assay used by Gorjao et al. [70] was ex vivo, whereas Dalli et al. [66] made use of the in vivo mouse peritonitis model. Further, there is a time-dependent factor when investigating the omega-3 fatty acids effect on neutrophil migration Arnardottir et al. [71] demonstrated in a peritonitis model that fewer neutrophils had migrated into the peritoneal cavity at 12 h, whereas at 48 h, more neutrophils had migrated into the peritoneal cavity compared with control mice.

### 3.2. Effects of Omega-3 Fatty Acids on the Phagocytic Capacity of Neutrophils

Omega-3 fatty acids have been shown to improve the phagocytic capacity in neutrophils in mice [72]. In vitro, adding DHA to extracted peritoneal neutrophils leads to a 35% increase in phagocytic capacity as well as a two-fold increase in fungicidal capacity [4]. Interestingly, EPA did not affect the phagocytic capacity in the same experiment. Polymorphonuclear leukocytes from goats incubated with either EPA or DHA had increased capacity to phagocytose *E. coli* compared with control [73]. This effect has partly been confirmed in humans too. Ten volunteers were given fish oil supplementation containing 26% EPA and 54% DHA daily for two months. Thereafter, the phagocytic capacity of the neutrophils in the blood was increased with 62% [70]. In another study, healthy volunteers were either given supplementation with corn oil (mostly linoleic acid, omega-6 fatty acids) or EPA. Interestingly, this led to a no effect on phagocytic capacity tested with *E. coli* in vitro [74]. This might indicate that the effect on phagocytic capacity is due to DHA.

### 3.3. Effects of Omega-3 Fatty Acids on the Production of Reactive Oxygen Species

Omega-3 fatty acids can influence neutrophil ROS production; however, the effect varies depending on animal model used as well as age (in humans). Both EPA and DHA increase the production of ROS in a dose-dependent manner in rat neutrophils, with a greater increase by EPA [4]. In goat polymorphonuclear leukocytes, the production of reactive oxygen species is decreased by DHA and not affected by EPA [73]. In humans, supplementation with 54% DHA and 26% EPA for two months increased ROS production in phorbol-myristate-acetate stimulated neutrophils [70]. However, since the supplementation contains both EPA and DHA, it is not obvious which fatty acid causes the effect on ROS production. Another experiment on humans was conducted by Rees et al. Healthy volunteers were either given supplementation with the linoleic-rich corn oil or EPA. Interestingly, EPA supplementation led to a decrease in ROS production in older men, but there was no change in younger men [74].

### 3.4. Other Aspects

Not only the function but also the number of neutrophils seems to be affected by omega-3 fatty acids. A diet rich in omega-3 PUFAs increases the frequency of neutrophils, as well as the number of CD117+ precursor cells, in bone marrow [72] and the frequency of neutrophils in the spleen and the liver in homeostatic conditions [75]. Dietary supplementation with fish oil also increases the number of neutrophils in the bone marrow and in the spleen of SMAD3 KO mice, with a more pronounced effect in male mice [76]. In humans, dietary supplementation of fish oil in healthy volunteers does not alter the number of circulating neutrophils [77].

The functions of neutrophils are not limited to migration, phagocytosis of pathogens and ROS production. They also secrete cytokines to orchestrate the immune response as well as cytotoxic proteins upon degranulation and they produce neutrophil extracellular traps (NETs) to immobilize pathogens [78]. To date, the reports investigating the effects of omega-3 fatty acids on these neutrophil functions are extremely scarce. Only two reports describe direct regulation of omega-3 fatty acids on neutrophil cytokine production. A report in 2013 demonstrated that EPA and DHA increase cytokine secretion (CXCL3, TNF-α and IL-1-β, respectively) in LPS-stimulated rat peritoneal neutrophils [4]. However, Capo et al. showed in 2015 that neutrophils extracted from the blood of sportsmen whose diet had been supplemented with DHA and EPA secreted lower amounts of IL-6 upon PMA stimulation [79]. A single report in 2018 investigated the effects of Resolvin D1 on NET production, i.e., in an abdominal aortic aneurysm mouse model Resolvin D1 decreased NET formation [80]. We were not able to find a single report investigating the effect of omega-3 fatty acids on neutrophil degranulation.

Finally, although lipid rafts have been shown to be important for neutrophil’s rolling [81], to our knowledge, not a single report has demonstrated a direct link between omega-3 fatty acids incorporation into the cellular membrane of neutrophils and its impact on lipid raft formation and neutrophil function.

## 4. Effects of Omega-3 Fatty Acids on T Cells

T cells are thymus-derived lymphocytes that recognize antigens presented by antigen presenting cells (APCs) through the T cell receptor (TCR). They comprise a heterogeneous group of cells with different immune properties, which makes their classification complex. T cells are classically classified into two main subsets, CD4+ T cells and CD8+ T cells, depending on their surface expression of CD4 or CD8 molecules, respectively. Both subsets differ on immune properties and functions. Whereas CD4+ T cells play major roles against bacterial infections, CD8 T cells mediate the immune response against viral infections. Additionally, T cells can be classified into helper (Th) and cytotoxic T cells. Th cells regulate the function of other immune cells whereas cytotoxic cells destroy virus-infected cells.

CD4+ helper T (Th) cells differentiate into different subgroups: Th1, Th2, Th17, and Th22 [82]. Each subgroup produces and secretes different cytokines in response to stimulation. Th1 are characterized by their secretion of IFN-γ, Th2 secrete IL-4, Th17 secrete IL-17A, IL17-F, IL-21 [83], and IL-22 and Th22 secrete IL-22.

Regulatory T cells (Tregs) suppress the activation of other immune cells such as Th1 CD4+ T cells, Th17 CD4+ T cells, CD8+ T cells, B cells, macrophages, or dendritic cells and maintain peripheral self-tolerance. The immunosuppressive effects of Tregs are exerted via the secretion of IL-10 and TGF-β [84].

Pro-inflammatory T cells are the cytotoxic CD8+ T cells and the CD4+ Th1 and Th17 cells. Th1 and Th17 cells are derived from CD4+ T cells upon stimulation with antigenic peptides and specific combination of cytokines [85]. Th17 cells are differentiated from naïve CD4+ T cells upon stimulation of IL-6 and TGF-β, which activate STAT3 and the transcription factor ROR-γt [86].

### 4.1. General Effects of Omega-3 Fatty Acids on T Cells

T cells are first activated by the interaction of the T cell receptor (TCR) with APCs such as macrophages or dendritic cells. Therefore, alterations in the activation of APCs by omega-3 fatty acids are the first mechanisms by which omega-3 fatty acids may modulate T cell activation in vivo [87,88]. However, direct effects of omega-3 fatty acids have also been described in the literature.

Evidence from multiple studies in vitro and in animal models concurs on a general suppressive effect of dietary omega-3 on T cell function [89,90]. In fact, omega-3 fatty acid supplementation has been shown to have beneficial effects in several T-mediated diseases such as autoimmune hepatitis [91] and asthma [92]. However, the modulatory effect of omega-3 fatty acids differs for each phenotypic subgroup of T cells.

No changes in the percentage of T cells in blood have been found in mice fed with a diet rich in omega-3 fatty acids [93], although an increase in Tregs and Th22 and a decrease in Th1, Th2, and Th17 cells was found in omega-3-fed mice after induction of colitis [93]. No alterations on the percentage of T cells or T cell populations have been found in blood in humans after supplementation with omega-3 fatty acids [77,94].

The effects of dietary omega-3 fatty acids on the abundance of T cells in the spleen is still not clear. Whereas Monk et al. reported an increase in the number of CD4+ and CD8+ T cells in the spleen of mice fed a diet rich in fish oil [95], we have recently showed that a similar diet does not induce any changes in the percentages of both populations in the spleen [75]. However, Monk et al. showed in a previous study that the omega-3-rich diet does not alter the abundance of Tregs, Th17 or Th1 cells in the spleen of mice [96].

### 4.2. Specific Effects of Omega-3 Fatty Acids on the Different Subgroups of T Cells

#### 4.2.1. CD4 T Cells

Metabolites derived from omega-3 fatty acids, i.e., SPMs, not only prevent differentiation of human CD4+ cells into Th1 cells, but also blunt the secretion of IFN-γ, IL-17, and IL-2 by human CD4+ T cells and TNF-α, IFN-γ, and IL-2 secretion by activated human CD8+ T cells [97], although, without inducing any changes in T cell viability or proliferation. Similarly, ex vivo activation of CD4+ T cells isolated from the spleen of mice fed a diet rich in omega-3 fatty acids is reduced in comparison with cells isolated from mice fed a normal diet [95]. This effect is mediated by an omega-3 fatty acid–induced decrease in the localization of signaling proteins and mitochondria at the immunological synapse [98,99]. In contrast to the case for human cells, however, omega-3 fatty acids decrease the proliferation of mouse CD4+ T cells [12,100].

Lipid rafts are important for the activation of CD4+ T cells [101]. Incorporation of omega-3 fatty acids into the membrane of CD4+ T cells from mice induces changes in the membrane domains organization both in mice [102] and human cells [103], which could account for the above mentioned effects of omega-3 fatty acids on CD4 T cell function.

#### 4.2.2. Th17 Cells

Research on multiple animal models has proved that omega-3 fatty acids blunt Th17 differentiation and activation. Treatment of CD4+ T cells isolated from human blood with anti-CD3 and anti-TNF-α induces the differentiation of cells towards Th17 cells in vitro. This increase in Th17 differentiation is prevented by the addition of EPA to the cells [104]. Similarly, CD4+ T cells differentiation into Th17 is blunted by omega-3 derived lipid mediators in human cells and in vivo in mouse models [97]. In wild-type mice fed a diet rich in omega-3 fatty acids and in the *fat-1* mouse model, which produces higher levels of endogenous omega-3 fatty acids, CD4+ T cells failed to activate Stat-3 in response to pro-Th17 signals compared to control mice [95,105].

In accordance to the decrease in the differentiation of CD4+ T cells into Th17 cells, omega-3 fatty acid supplementation reduces IL-17 plasma levels in children with asthma [92]. Limitation of IL-17 plasma levels by dietary omega-3 fatty acids improves the severity of autoimmune uveitis in mice [106].

#### 4.2.3. Regulatory T Cells

Due to their immune-regulatory properties, Tregs play important roles in many immune diseases. As such, the effects of omega-3 fatty acids on Tregs have been the focus of more extensive investigation than other subtypes of T cells. Dietary omega-3 fatty acids promote the accumulation and proliferation of Tregs in the liver [107], spleen [108] and in adipose tissue [87] of wild-type mice. Also, *fat-1* mice present higher numbers of splenic Tregs due to enhanced differentiation towards Treg phenotype due to the increased production of endogenous omega-3 fatty acids [109].

Both EPA and DHA promote the proliferation in vitro of CD4+ CD25+ cells isolated from mice into Tregs [19,107]. In vivo, differentiation of T cells into Tregs has been found to be enhanced by the omega-3 fatty acid–mediated increase in M2 macrophages, as M2 macrophages directly induce Treg differentiation [87,110].

The promotion of CD4+ T cell differentiation into Tregs by dietary omega-3 fatty acids has been successfully employed to reduce the severity of diseases such as arthritis and atopic dermatitis in mouse models [109,110].

## 5. Effects of Omega-3 Fatty Acids on B Cells

B cells are, together with T cells, the main lymphocytes of the adaptive branch of the immune response. Although the main classical function attributed to B cells is the production of antibodies, recent studies highlight B cell capacity of response to innate immune stimuli, such as PAMPs, and their important role in the regulation of the immune response via antigen presentation and cytokine production.

B cells originate in the bone marrow from hematopoietic stem cells (HSCs). HSCs differentiate into pro-B cells, the state at which heavy chain immunoglobulin rearrangement starts, and subsequently to pre-B cells, when the light chain immunoglobulin rearrangement starts. Once the immunoglobulin rearrangement is completed, the B cell receptor is fully functional, and B cells become immature B cells. After being tested for auto-reactivity, immature B cells migrate to the spleen, where they undergo further differentiation into follicular B cells or marginal zone B cells, via two transitional differentiation steps known as transitional type 1 B cells and transitional type 2 B cells [111].

Additionally, one type of innate-like B cells, known as B1 cells, has been characterized, [112]. B1 cells can function as antigen presenting cells and produce antibodies like conventional B cells (also known as B2 cells) but are more often present in peripheral tissues like the peritoneal cavity than in blood [113].

### 5.1. Effects of Omega-3 Fatty Acids on B Cell Populations

A diet rich in omega-3 fatty acids exerts alterations in the percentages of the different B cell populations in different tissues in mice. Mice fed a diet rich in omega-3 fatty acids from fish oil present a decreased amount of naïve B cells and mature B cells in the bone marrow but no changes in the percentages of pre-B cells or pro-B cells [114]. However, a diet rich in omega-3 fatty acids from fish oil does not alter the percentage of B1 or B2 cells in the peritoneal cavity [115,116]. In the spleen, the proportion between the different B cell populations is altered by dietary omega-3 fatty acids from fish oil while the total number of splenic B cells remains the same [75,117,118]. Specifically, mice fed a diet enriched with fish oil omega-3 have increased numbers of transitional type 1 B cells in the spleen [114,115], whereas mice fed diet enriched with DHA or with EPA present higher numbers of both transitional type 1 B and transitional type 2 B cell populations [117]. Finally, dietary EPA supplementation increases the amount of total B cells in the adipose tissue of mice [87].

### 5.2. Effects of Omega-3 Fatty Acids on B Cell Activation and Antibody Production

B cells can be activated by both the interaction with other immune cells, such as T cells or neutrophils, or by directly binding pathogen-associated molecular patterns (PAMPs) [119]. In response to stimulation, B cells increase the surface expression of several molecules, including CD80, CD86, MCH class II, and CD40 [120].

DHA and EPA have a negative effect on the immune activation of B cells extracted from human PBMCs and treated ex vivo. EPA and DHA decrease anti-CD-40-induced p50 nuclear translocation, phosphorylation of Stat6 and secretion of IL-6 [121]. The DHA-derived metabolite 17-hDHA decreases IL-6 and IL-10 secretion by human B cells upon stimulation with TLR agonist CpG ODN 2395 and anti-IgM [122].

However, current results regarding the effect of omega-3 fatty acids on B cell activation on mouse present some contradictions. On one hand, B cells isolated from the spleen of mice previously fed with diets rich in omega-3 fatty acids secrete less IL-6 upon ex vivo stimulation with LPS [14,123] and less IL-12 upon ex vivo stimulation with T cells [124]. In these conditions, omega-3 fatty acids did not alter B cell activation marker expression, such as CD80, CD69, MHC II, or CD80. On the other hand, in vivo analysis of splenic B cell activation in mice revealed that a diet rich in omega-3 fatty acids increases levels of CD69, MHC II, and CD11c [14,123]. Interestingly, whether the analysis of the activation of B cells was done after ex vivo incubation with omega-3 or in vivo in mice fed with omega-3-rich diets, omega-3 fatty acids does not alter the proportion of B cells that get activated [123,124].

Studies regarding the effects of omega-3 fatty acids on the activation of B cell lymphoblasts confirm the blunting effect on B cell activation by omega-3 fatty acids, although the data is scarce. Shaikh et al. reported that JY B lymphoblasts are less susceptible to be lysed by alloreactive CD8 T cells after incubation with DHA due to decreased MHC I expression and decreased conjugate formation [125]. Verlengia et al. showed that Raji cells stimulated with Concanavalin A secreted less IL-10, TNF-α, and IFN-γ after incubation with EPA or DHA [126].

Although most studies to date conclude that omega-3 fatty acids reduce B cell activation to a certain extent, there is still some controversy in the field. A possible source of variability in these experiments is the fish oil added to the mice diet as a source of omega-3 fatty acids, as different fish oils have different effects on B cell activation even if they induce a similar accumulation of omega-3 fatty acids on the cellular membrane [127].

Interestingly, despite most of the current evidence pointing to the dampening effect of omega-3 fatty acids on B cell activation, both EPA and DHA increase IgM production by B cells by increasing the number of antibody-producing cells in mouse and human [114,117,122]. This effect is quite specific for IgM since omega-3 fatty acids do not alter B cell production of IgA, IgG, or IgD [114,121].

## 6. Effects of Omega-3 Fatty Acids on Other Immune Cells

Less extensive investigation has been performed on the effects of omega-3 fatty acids on other immune cells besides macrophages, neutrophils, T cells, and B cells. In the following section, we summarize the main findings regarding the alterations on other immune cells functions caused by omega-3 fatty acids.

### 6.1. Dendritic Cells

Dendritic cells play an important role in the activation and regulation of the adaptive immune system thanks to their role as APCs [128]. It is in this context that the effects of omega-3 fatty acids on dendritic cells has mainly been investigated. The omega-3 fatty acids DHA and EPA cause a down regulation of the MHC II and co-stimulatory molecules in the surface of mouse and human dendritic cells [129,130,131,132]. As a direct consequence, the activation of T cells by omega-3 fatty acids–treated dendritic cells is severely impaired [88,131,133,134,135].

### 6.2. Natural Killer Cells

Natural killer (NK) cells are innate lymphocytes that mediate the immune response against tumor and viruses. In contrast with cytotoxic CD8+ T cells, NK cells do not require prior contact with an antigen for their activation [136]. To date, little is known regarding the effects of omega-3 fatty acids on NK function, and the current evidence is often contradictory. Whereas it was reported that dietary DHA promotes activation of splenic NK cells in mouse [137], the opposite was found in a mouse model of influenza infection [138]. In humans, dietary supplementation with EPA-rich oil did not alter the number of circulating NK cells [94]. However, a similar study two years later found that dietary supplementation of DHA and EPA lowered the percentage of NK cells in blood [77]. The effects of omega-3 fatty acid supplementation on NK cells in humans might be age-dependent, since EPA decreases NK cell activity in humans older than 55 years [139].

### 6.3. Mast Cells

Mast cells are innate immune cells that have been classically associated with allergic processes. They bind IgE secreted by B cells through their high affinity IgE receptor (FcϵRI receptor). The interaction of an antigen with the IgE-FcϵRI receptor complex initiates a signaling cascade that culminates with mast cell degranulation [140]. Omega-3 fatty acids decrease IgE-mediated activation of mast cells in several animal models and in human cells [141,142,143]. The dampening effect of omega-3 fatty acids on mast cell activation has been employed to decrease the severity of mast cell-associated diseases such as allergy or atopic dermatitis [141,144,145,146]. However, fish oil supplementation in asthmatic patients failed to ameliorate their symptoms [147].

### 6.4. Basophils

Basophils are the least frequent granulocyte immune cell type present in blood. Their function and immunological properties are similar to those of mast cells, therefore they also play important roles in allergy and asthma [148]. Treatment of basophilic leukemia cells with EPA and DHA reduces basophil activation in vitro [149,150]. In healthy humans, dietary supplementation with omega-3 fatty acids decreases circulating levels of basophils [151]. In asthmatic patients, dietary supplementation with botanical omega-3 fatty acids decreases basophil production of leukotrienes ex vivo [152].

### 6.5. Eosinophils

Together with neutrophils and basophils, eosinophils are part of the granulocyte family of innate immune cells. Similarly to mast cells and basophils, they mediate immune reactions in allergic diseases [153]. Oral administration of omega-3 fatty acids decreases the recruitment of eosinophils into the airways in eosinophilic airway inflammation murine models as well as eosinophil infiltration in a murine atopic dermatitis model, murine allergic conjunctivitis model and a rat skin allergy model [141,154,155,156,157]. However, eosinophil numbers in the adipose tissue are increased upon EPA supplementation in lean and obese mice [87]. In humans, supplementation with omega-3 fatty acid decreases the amount of circulating eosinophils in healthy volunteers [151], but it failed to induce any changes in sputum eosinophilic count in asthmatic patients [147]. The suppressive effect of omega-3 fatty acids on eosinophil tissue infiltration could be related to the inhibitory effect on eosinophil proliferation and migration by DHA found in vitro [158,159].

## 7. Concluding Remarks

In the present review, we have summarized the main findings regarding the immunomodulatory effects of omega-3 fatty acids on the different cells that conform the immune system (summarized in Table 1). To our knowledge, from all the immune cells investigated to date, none of them has been found to be inert to dietary omega-3 fatty acids. Generally, ALA, DHA, and EPA exert an inhibitory effect on the activation of immune cells from both the innate and the adaptive branch. Interestingly, some specific immune functions are promoted by dietary omega-3 fatty acids in specific immune cell types, i.e., phagocytosis by macrophages and neutrophils or Treg differentiation, suggesting that omega-3 fatty acids do not act as unspecific immune-repressors.

Variations between different reports may lay on the different protocols for the incorporation of omega-3 fatty acids in in vitro cell culture, for example different concentrations or incubation times, or in vivo, e.g., different amounts of omega-3 fatty acids, proportions between omega-3 and omega-6 in the diet, duration of the supplementation, etc. Moreover, in order to correctly interpret the results from the investigations regarding the effects of dietary omega-3 fatty acids in vivo, the differences in fatty acid metabolism between different animal models should be taken into consideration. For example, dietary supplementation of omega-3 fatty acids increases omega-3 fatty acid blood levels more efficiently compared with employing the *fat-1* transgenic mouse model, particularly for EPA [160]. Further, findings in mouse models may not be directly translated into humans since both organisms have differences at the immunological and metabolic levels. Often, dietary supplementation of omega-3 fatty acids in humans contains lower levels of EPA or DHA and is maintained for shorter time-periods compared with dietary supplementation of omega-3 fatty acids in mouse experiments. Additionally, while mouse diet is completely under the control of the investigator, human diet is more complex and heterogeneous, which could interfere with the effects of omega-3 fatty acid supplementation. To date, two main indices have been used to quantify the enrichment of the diet on omega-3 fatty acids: the n-3/n-6 ratio and the omega-3 index. Recent reports suggest a new index, named PUFA balance, which may improve the evaluation of omega-3 supplementation in humans and animal models [161,162].

Although great progress has been made in our understanding of the effects of omega-3 fatty acids on the cells of the immune system, we still do not fully comprehend the precise mechanisms by which omega-3 fatty acids exert their immunoregulatory effects. How many of the effects exerted by omega-3 fatty acids rely on their localization on the cellular membrane? How many of them rely on the activation of transcription factors? Are the transcription factors activated by omega-3 fatty acids the same in all immune cells?

Omega-3 fatty acids ameliorate symptoms in several animal disease models, such as sepsis or autoimmune hepatitis, and have been tested in clinical trials with positive outcome. Getting detailed knowledge about the particular direct effects of omega-3 fatty acids on the different cells of the immune system will allow future investigators and clinicians to further optimize and implement omega-3 supplementation for the treatment of multiple diseases.

## Figures and Tables

**Table 1:** Summary of the properties of omega-3 fatty acids and their mediators on the different cells of the immune system including main references.

Cell Type	Effect	References
Macrophages	↓ cytokines	Cytokine production and secretion [29,30,31,32,33,34,35,36,37,39,40,41,42] Signaling [28,38,44,45,46,47]
	↑ polarization towards M2 phenotype	[29,30,49] Stroke [50] Atopic dermatitis [51]
	↑ phagocytosis	Zymosan [29] *R.equi, P.aeruginosa* [52] *E.coli* [53] Apoptotic cells [29]
Neutrophils	↑ production of pro-resolving mediators	[61,62]
	↓ migration	[63,64,65,66,67,68,71]
	↑ phagocytosis	Particles [72] *C. albicans* [4] *E.coli* [73,74] Zymosan [70]
	↔ ROS production	Rat [4] Goat [73] Human [70,74]
	↑ frequency	[72,75,76]
Eosinophils	↓ infiltration	Airway inflammation [155] Skin [141,156] Allergy [154,157]
Basophils	↓ activation	[149,150,152]
Dendritic cells	↓ antigen presentation	[88,129,130,131,133,134]
NK cells	↔ activation	[137,138,139]
Mast cells	↓ activation	[141,142,143,144,145,146]
T cells	↓ activation	General effects [87,88,89,90,91,92] CD4+ T cells [95,97,98,99] Th17 T cells [92,95,97,104,105,106]
	↑ Treg differentiation	[19,87,107,108,109,110]
B cells	↔ activation	Human [121,122,125,126] Mouse [14,123,124]
	↑ IgM production	[114,117,121,122]

↓ indicates a decrease, ↑ indicates an increase, ↔ indicates contradictive evidence.

## Abbreviations

ALA	Alpha (α)-linolenic acid
DHA	docosahexaenoic acid
EPA	eicosapentaenoic acid
PAMPS	pathogen-associated molecular patterns
PUFAs	polyunsaturated fatty acids
ROS	reactive oxygen species
SPMs	pro-resolving mediators

## References

[1] Sokol C.L., Luster A.D. (2015). The chemokine system in innate immunity. Cold Spring Harb. Perspect Biol..

[2] Iwasaki A., Medzhitov R. (2015). Control of adaptive immunity by the innate immune system. Nat. Immunol..

[3] Wu D., Lewis E.D., Pae M., Meydani S.N. (2018). Nutritional Modulation of Immune Function: Analysis of Evidence, Mechanisms, and Clinical Relevance. Front. Immunol..

[4] Paschoal V.A., Vinolo M.A., Crisma A.R., Magdalon J., Curi R. (2013). Eicosapentaenoic (EPA) and docosahexaenoic (DHA) acid differentially modulate rat neutrophil function in vitro. Lipids.

[5] Calder P.C. (2014). Metabolic benefits of marine n-3 fatty acids demonstrated in nonhuman primates. J. Nutr..

[6] Cholewski M., Tomczykowa M., Tomczyk M. (2018). A Comprehensive Review of Chemistry, Sources and Bioavailability of Omega-3 Fatty Acids. Nutrients.

[7] Calder P.C. (2016). Docosahexaenoic Acid. Ann. Nutr. Metab..

[8] Wiktorowska-Owczarek A., Berezinska M., Nowak J.Z. (2015). PUFAs: Structures, Metabolism and Functions. Adv. Clin. Exp. Med..

[9] Metherel A.H., Lacombe R.J.S., Chouinard-Watkins R., Hopperton K.E., Bazinet R.P. (2018). Complete assessment of whole-body n-3 and n-6 PUFA synthesis-secretion kinetics and DHA turnover in a rodent model. J. Lipid Res..

[10] Rodrigues F.G., Campos J.B., Silva G.D., Wexner S.D. (2016). Endoscopic ultrasound in the diagnosis of foreign bodies of the colon and rectum. Rev. Assoc. Med. Bras..

[11] Calder P.C. (2005). Polyunsaturated fatty acids and inflammation. Biochem Soc. Trans..

[12] Yessoufou A., Ple A., Moutairou K., Hichami A., Khan N.A. (2009). Docosahexaenoic acid reduces suppressive and migratory functions of CD4(+)CD25(+) regulatory T-cells. J. Lipid Res..

[13] Sorensen L.S., Thorlacius-Ussing O., Rasmussen H.H., Lundbye-Christensen S., Calder P.C., Lindorff-Larsen K., Schmidt E.B. (2014). Effects of perioperative supplementation with omega-3 fatty acids on leukotriene B(4) and leukotriene B(5) production by stimulated neutrophils in patients with colorectal cancer: A randomized, placebo-controlled intervention trial. Nutrients.

[14] Gurzell E.A., Teague H., Harris M., Clinthorne J., Shaikh S.R., Fenton J.I. (2013). DHA-enriched fish oil targets B cell lipid microdomains and enhances ex vivo and in vivo B cell function. J. Leukoc. Biol..

[15] Hashimoto M., Hossain S., Waisundara V. (2018). Fatty Acids: From Membrane Ingredients to Signaling Molecules. Biochemistry and Health Benefits of Fatty Acids.

[16] Yates C.M., Calder P.C., Ed Rainger G. (2014). Pharmacology and therapeutics of omega-3 polyunsaturated fatty acids in chronic inflammatory disease. Pharmacol. Ther..

[17] Fritsche K. (2006). Fatty acids as modulators of the immune response. Annu. Rev. Nutr..

[18] Husson M.O., Ley D., Portal C., Gottrand M., Hueso T., Desseyn J.L., Gottrand F. (2016). Modulation of host defence against bacterial and viral infections by omega-3 polyunsaturated fatty acids. J. Infect..

[19] Bi X., Li F., Liu S., Jin Y., Zhang X., Yang T., Dai Y., Li X., Zhao A.Z. (2017). omega-3 polyunsaturated fatty acids ameliorate type 1 diabetes and autoimmunity. J. Clin. Invest..

[20] Gordon S., Pluddemann A., Estrada F.M. (2014). Macrophage heterogeneity in tissues: phenotypic diversity and functions. Immunol. Rev..

[21] Murray P.J. (2017). Macrophage Polarization. Annu Rev. Physiol..

[22] Magrum L.J., Johnston P.V. (1983). Modulation of prostaglandin synthesis in rat peritoneal macrophages with omega-3 fatty acids. Lipids.

[23] Schroit A.J., Gallily R. (1979). Macrophage fatty acid composition and phagocytosis: effect of unsaturation on cellular phagocytic activity. Immunology.

[24] Allam-Ndoul B., Guenard F., Barbier O., Vohl M.C. (2017). A Study of the Differential Effects of Eicosapentaenoic Acid (EPA) and Docosahexaenoic Acid (DHA) on Gene Expression Profiles of Stimulated Thp-1 Macrophages. Nutrients.

[25] Roessler C., Kuhlmann K., Hellwing C., Leimert A., Schumann J. (2017). Impact of Polyunsaturated Fatty Acids on miRNA Profiles of Monocytes/Macrophages and Endothelial Cells-A Pilot Study. Int. J. Mol. Sci..

[26] Calder P.C. (2011). Fatty acids and inflammation: The cutting edge between food and pharma. Eur. J. Pharmacol..

[27] Oh D.Y., Talukdar S., Bae E.J., Imamura T., Morinaga H., Fan W.Q., Li P.P., Lu W.J., Watkins S.M., Olefsky J.M. (2010). GPR120 Is an Omega-3 Fatty Acid Receptor Mediating Potent Anti-inflammatory and Insulin-Sensitizing Effects. Cell.

[28] Mildenberger J., Johansson I., Sergin I., Kjobli E., Damas J.K., Razani B., Flo T.H., Bjorkoy G. (2017). N-3 PUFAs induce inflammatory tolerance by formation of KEAP1-containing SQSTM1/p62-bodies and activation of NFE2L2. Autophagy.

[29] Chang H.Y., Lee H.N., Kim W., Surh Y.J. (2015). Docosahexaenoic acid induces M2 macrophage polarization through peroxisome proliferator-activated receptor gamma activation. Life Sci..

[30] Titos E., Rius B., Gonzalez-Periz A., Lopez-Vicario C., Moran-Salvador E., Martinez-Clemente M., Arroyo V., Claria J. (2011). Resolvin D1 and Its Precursor Docosahexaenoic Acid Promote Resolution of Adipose Tissue Inflammation by Eliciting Macrophage Polarization toward an M2-Like Phenotype. J. Immun..

[31] Yan Y., Jiang W., Spinetti T., Tardivel A., Castillo R., Bourquin C., Guarda G., Tian Z., Tschopp J., Zhou R. (2013). Omega-3 fatty acids prevent inflammation and metabolic disorder through inhibition of NLRP3 inflammasome activation. Immunity.

[32] Jin J.F., Lu Z.Y., Li Y.C., Cowart L.A., Lopes-Virella M.F., Yan H. (2018). Docosahexaenoic acid antagonizes the boosting effect of palmitic acid on LPS inflammatory signaling by inhibiting gene transcription and ceramide synthesis. PLoS ONE.

[33] Kumar N., Gupta G., Anilkumar K., Fatima N., Karnati R., Reddy G.V., Giri P.V., Reddanna P. (2016). 15-Lipoxygenase metabolites of alpha-linolenic acid, [13-(S)-HPOTrE and 13-(S)-HOTrE], mediate anti-inflammatory effects by inactivating NLRP3 inflammasome. Sci. Rep..

[34] Honda K.L., Lamon-Fava S., Matthan N.R., Wu D.Y., Lichtenstein A.H. (2015). Docosahexaenoic acid differentially affects TNF alpha and IL-6 expression in LPS-stimulated RAW 264.7 murine macrophages. Prostaglandins Leukot. Essent. Fat. Acids.

[35] Allam-Ndoul B., Guenard F., Barbier O., Vohl M.C. (2017). Effect of different concentrations of omega-3 fatty acids on stimulated THP-1 macrophages. Genes Nutr..

[36] Takashima A., Fukuda D., Tanaka K., Higashikuni Y., Hirata Y., Nishimoto S., Yagi S., Yamada H., Soeki T., Wakatsuki T. (2016). Combination of n-3 polyunsaturated fatty acids reduces atherogenesis in apolipoprotein E-deficient mice by inhibiting macrophage activation. Atherosclerosis.

[37] Liu Y., Chen L.Y., Sokolowska M., Eberlein M., Alsaaty S., Martinez-Anton A., Logun C., Qi H.Y., Shelhamer J.H. (2014). The fish oil ingredient, docosahexaenoic acid, activates cytosolic phospholipase A(2) via GPR120 receptor to produce prostaglandin E(2) and plays an anti-inflammatory role in macrophages. Immunology.

[38] Honda K.L., Lamon-Fava S., Matthan N.R., Wu D.Y., Lichtenstein A.H. (2015). EPA and DHA Exposure Alters the Inflammatory Response but not the Surface Expression of Toll-like Receptor 4 in Macrophages. Lipids.

[39] Song Z.X., Xia H., Yang L.G., Wang S.K., Sun G.J. (2018). Lowering the n-6/n-3 PUFAs ratio inhibits the formation of THP-1 macrophage-derived foam cell. Lipids Health Dis..

[40] Schoeniger A., Adolph S., Fuhrmann H., Schumann J. (2011). The Impact of Membrane Lipid Composition on Macrophage Activation in the Immune Defense against Rhodococcus equi and Pseudomonas aeruginosa. Int. J. Mol. Sci..

[41] Iverson C., Bacong A., Liu S., Baumgartner S., Lundstrom T., Oscarsson J., Miner J.N. (2018). Omega-3-carboxylic acids provide efficacious anti-inflammatory activity in models of crystal-mediated inflammation. Sci. Rep..

[42] Williams-Bey Y., Boularan C., Vural A., Huang N.N., Hwang I.Y., Shan-Shi C., Kehrl J.H. (2014). Omega-3 free fatty acids suppress macrophage inflammasome activation by inhibiting NF-kappaB activation and enhancing autophagy. PLoS ONE.

[43] Yan Z.Q., Hansson G.K. (2007). Innate immunity, macrophage activation, and atherosclerosis. Immunol Rev..

[44] Schoeniger A., Fuhrmann H., Schumann J. (2016). LPS- or Pseudomonas aeruginosa-mediated activation of the macrophage TLR4 signaling cascade depends on membrane lipid composition. PeerJ.

[45] Hellwing C., Schoeniger A., Roessler C., Leimert A., Schumann J. (2018). Lipid raft localization of TLR2 and its co-receptors is independent of membrane lipid composition. PeerJ.

[46] Lee J.Y., Plakidas A., Lee W.H., Heikkinen A., Chanmugam P., Bray G., Hwang D.H. (2003). Differential modulation of Toll-like receptors by fatty acids: preferential inhibition by n-3 polyunsaturated fatty acids. J. Lipid Res..

[47] Sung J., Jeon H., Kim I.H., Jeong H.S., Lee J. (2017). Anti-Inflammatory Effects of Stearidonic Acid Mediated by Suppression of NF-kappa B and MAP-Kinase Pathways in Macrophages. Lipids.

[48] Marques-Rocha J.L., Garcia-Lacarte M., Samblas M., Bressan J., Martinez J.A., Milagro F.I. (2018). Regulatory roles of miR-155 and let-7b on the expression of inflammation-related genes in THP-1 cells: effects of fatty acids. J. Physiol. Biochem..

[49] Ohue-Kitano R., Yasuoka Y., Goto T., Kitamura N., Park S.B., Kishino S., Kimura I., Kasubuchi M., Takahashi H., Li Y.J. (2018). alpha-Linolenic acid-derived metabolites from gut lactic acid bacteria induce differentiation of anti-inflammatory M2 macrophages through G protein-coupled receptor 40. Faseb J..

[50] Cai W., Liu S.X., Hu M.Y., Sun X.B., Qiu W., Zheng X.M., Hu X.M., Lu Z.Q. (2018). Post-stroke DHA Treatment Protects Against Acute Ischemic Brain Injury by Skewing Macrophage Polarity Toward the M2 Phenotype. Transl. Stroke Res..

[51] Haitz K.A., Anandasabapathy N. (2015). Docosahexaenoic Acid alleviates atopic dermatitis in mice by generating T regulatory cells and m2 macrophages. J. Invest. Dermatol.

[52] Adolph S., Fuhrmann H., Schumann J. (2012). Unsaturated Fatty Acids Promote the Phagocytosis of P-aeruginosa and R-equi by RAW264.7 Macrophages. Curr. Microbiol..

[53] Davidson J., Kerr A., Guy K., Rotondo D. (1998). Prostaglandin and fatty acid modulation of Escherichia coli O157 phagocytosis by human monocytic cells. Immunology.

[54] Hellwing C., Tigistu-Sahle F., Fuhrmann H., Kakela R., Schumann J. (2018). Lipid composition of membrane microdomains isolated detergent-free from PUFA supplemented RAW264.7 macrophages. J. Cell. Physiol..

[55] Athens J.W., Haab O.P., Raab S.O., Mauer A.M., Ashenbrucker H., Cartwright G.E., Wintrobe M.M. (1961). Leukokinetic studies. IV. The total blood, circulating and marginal granulocyte pools and the granulocyte turnover rate in normal subjects. J. Clin. Invest..

[56] Athens J.W., Raab S.O., Haab O.P., Mauer A.M., Ashenbrucker H., Cartwright G.E., Wintrobe M.M. (1961). Leukokinetic studies. III. The distribution of granulocytes in the blood of normal subjects. J. Clin. Invest..

[57] Summers C., Rankin S.M., Condliffe A.M., Singh N., Peters A.M., Chilvers E.R. (2010). Neutrophil kinetics in health and disease. Trends Immunol..

[58] Kolaczkowska E., Kubes P. (2013). Neutrophil recruitment and function in health and inflammation. Nat. Rev. Immunol..

[59] Puga I., Cols M., Barra C.M., He B., Cassis L., Gentile M., Comerma L., Chorny A., Shan M., Xu W. (2012). B cell-helper neutrophils stimulate the diversification and production of immunoglobulin in the marginal zone of the spleen. Nat. Immunol..

[60] Veno S.K., Nielsen M.R., Lundbye-Christensen S., Schmidt E.B., Handberg A. (2013). The effect of low-dose marine n-3 fatty acids on plasma levels of sCD36 in overweight subjects: a randomized, double-blind, placebo-controlled trial. Mar. Drugs.

[61] Serhan C.N., Chiang N., Dalli J., Levy B.D. (2014). Lipid mediators in the resolution of inflammation. Cold Spring Harb Perspect Biol..

[62] Serhan C.N. (2014). Pro-resolving lipid mediators are leads for resolution physiology. Nature.

[63] Jones C.N., Dalli J., Dimisko L., Wong E., Serhan C.N., Irimia D. (2012). Microfluidic chambers for monitoring leukocyte trafficking and humanized nano-proresolving medicines interactions. Proc. Natl. Acad. Sci. USA.

[64] Krishnamoorthy S., Recchiuti A., Chiang N., Yacoubian S., Lee C.H., Yang R., Petasis N.A., Serhan C.N. (2010). Resolvin D1 binds human phagocytes with evidence for proresolving receptors. Proc. Natl. Acad. Sci. USA.

[65] Tull S.P., Yates C.M., Maskrey B.H., O’Donnell V.B., Madden J., Grimble R.F., Calder P.C., Nash G.B., Rainger G.E. (2009). Omega-3 Fatty acids and inflammation: novel interactions reveal a new step in neutrophil recruitment. PLoS Biol..

[66] Dalli J., Winkler J.W., Colas R.A., Arnardottir H., Cheng C.Y., Chiang N., Petasis N.A., Serhan C.N. (2013). Resolvin D3 and aspirin-triggered resolvin D3 are potent immunoresolvents. Chem. Biol..

[67] Serhan C.N., Yang R., Martinod K., Kasuga K., Pillai P.S., Porter T.F., Oh S.F., Spite M. (2009). Maresins: novel macrophage mediators with potent antiinflammatory and proresolving actions. J. Exp. Med..

[68] Deng B., Wang C.W., Arnardottir H.H., Li Y., Cheng C.Y., Dalli J., Serhan C.N. (2014). Maresin biosynthesis and identification of maresin 2, a new anti-inflammatory and pro-resolving mediator from human macrophages. PLoS ONE.

[69] Serhan C.N., Fredman G., Yang R., Karamnov S., Belayev L.S., Bazan N.G., Zhu M., Winkler J.W., Petasis N.A. (2011). Novel proresolving aspirin-triggered DHA pathway. Chem. Biol..

[70] Gorjao R., Verlengia R., Lima T.M., Soriano F.G., Boaventura M.F., Kanunfre C.C., Peres C.M., Sampaio S.C., Otton R., Folador A. (2006). Effect of docosahexaenoic acid-rich fish oil supplementation on human leukocyte function. Clin. Nutr..

[71] Arnardottir H.H., Freysdottir J., Hardardottir I. (2013). Dietary fish oil increases the proportion of a specific neutrophil subpopulation in blood and total neutrophils in peritoneum of mice following endotoxin-induced inflammation. J. Nutr. Biochem..

[72] Svahn S.L., Ulleryd M.A., Grahnemo L., Stahlman M., Boren J., Nilsson S., Jansson J.O., Johansson M.E. (2016). Dietary Omega-3 Fatty Acids Increase Survival and Decrease Bacterial Load in Mice Subjected to Staphylococcus aureus-Induced Sepsis. Infect Immun..

[73] Pisani L.F., Lecchi C., Invernizzi G., Sartorelli P., Savoini G., Ceciliani F. (2009). In vitro modulatory effect of omega-3 polyunsaturated fatty acid (EPA and DHA) on phagocytosis and ROS production of goat neutrophils. Vet. Immunol. Immunopathol..

[74] Rees D., Miles E.A., Banerjee T., Wells S.J., Roynette C.E., Wahle K.W., Calder P.C. (2006). Dose-related effects of eicosapentaenoic acid on innate immune function in healthy humans: a comparison of young and older men. Am. J. Clin. Nutr..

[75] Svahn S.L., Gutierrez S., Ulleryd M.A., Nookaew I., Osla V., Beckman F., Nilsson S., Karlsson A., Jansson J.O., Johansson M.E. (2019). Dietary polyunsaturated fatty acids promote neutrophil accumulation in spleen by altering chemotaxis and delaying cell death. Infect. Immun..

[76] Duriancik D.M., Comstock S.S., Langohr I.M., Fenton J.I. (2015). High levels of fish oil enhance neutrophil development and activation and influence colon mucus barrier function in a genetically susceptible mouse model. J. Nutr. Biochem..

[77] Mukaro V.R., Costabile M., Murphy K.J., Hii C.S., Howe P.R., Ferrante A. (2008). Leukocyte numbers and function in subjects eating n-3 enriched foods: selective depression of natural killer cell levels. Arthritis Res. Ther..

[78] Rosales C. (2018). Neutrophil: A Cell with Many Roles in Inflammation or Several Cell Types?. Front. Physiol..

[79] Capo X., Martorell M., Sureda A., Tur J.A., Pons A. (2015). Effects of docosahexaenoic supplementation and in vitro vitamin C on the oxidative and inflammatory neutrophil response to activation. Oxid Med. Cell Longev..

[80] Spinosa M., Su G., Salmon M.D., Lu G., Cullen J.M., Fashandi A.Z., Hawkins R.B., Montgomery W., Meher A.K., Conte M.S. (2018). Resolvin D1 decreases abdominal aortic aneurysm formation by inhibiting NETosis in a mouse model. J. Vasc. Surg..

[81] Xu R. (2015). Important Bioactive Properties of Omega-3 Fatty Acids. Ital. J. Food Sci..

[82] Gagliani N., Huber S. (2017). Basic Aspects of T Helper Cell Differentiation. Methods Mol. Biol..

[83] Korn T., Bettelli E., Oukka M., Kuchroo V.K. (2009). IL-17 and Th17 Cells. Annu. Rev. Immunol..

[84] Sharabi A., Tsokos M.G., Ding Y., Malek T.R., Klatzmann D., Tsokos G.C. (2018). Regulatory T cells in the treatment of disease. Nat. Rev. Drug Discov..

[85] Raphael I., Nalawade S., Eagar T.N., Forsthuber T.G. (2015). T cell subsets and their signature cytokines in autoimmune and inflammatory diseases. Cytokine.

[86] Bettelli E., Carrier Y.J., Gao W.D., Korn T., Strom T.B., Oukka M., Weiner H.L., Kuchroo V.K. (2006). Reciprocal developmental pathways for the generation of pathogenic effector T(H)17 and regulatory T cells. Nature.

[87] Onodera T., Fukuhara A., Shin J., Hayakawa T., Otsuki M., Shimomura I. (2017). Eicosapentaenoic acid and 5-HEPE enhance macrophage-mediated Treg induction in mice. Sci. Rep..

[88] Carlsson J.A., Wold A.E., Sandberg A.S., Ostman S.M. (2015). The Polyunsaturated Fatty Acids Arachidonic Acid and Docosahexaenoic Acid Induce Mouse Dendritic Cells Maturation but Reduce T-Cell Responses In Vitro. PLoS ONE.

[89] Endres S., Meydani S.N., Ghorbani R., Schindler R., Dinarello C.A. (1993). Dietary supplementation with n-3 fatty acids suppresses interleukin-2 production and mononuclear cell proliferation. J. Leukoc. Biol..

[90] Yaqoob P., Newsholme E.A., Calder P.C. (1994). The effect of dietary lipid manipulation on rat lymphocyte subsets and proliferation. Immunology.

[91] Li Y.L., Tang Y., Wang S.J., Zhou J., Zhou J., Lu X., Bai X.C., Wang X.Y., Chen Z.L., Zuo D.M. (2016). Endogenous n-3 Polyunsaturated Fatty Acids Attenuate T Cell-Mediated Hepatitis via Autophagy Activation. Front. Immunol..

[92] Farjadian S., Moghtaderi M., Kalani M., Gholami T., Teshnizi S.H. (2016). Effects of omega-3 fatty acids on serum levels of T-helper cytokines in children with asthma. Cytokine.

[93] Huang C.H., Hou Y.C., Pai M.H., Yeh C.L., Yeh S.L. (2017). Dietary omega-6/omega-3 Polyunsaturated Fatty Acid Ratios Affect the Homeostasis of Th/Treg Cells in Mice With Dextran Sulfate Sodium-Induced Colitis. JPEN J. Parenter. Enter. Nutr..

[94] Miles E.A., Banerjee T., Wells S.J., Calder P.C. (2006). Limited effect of eicosapentaenoic acid on T-lymphocyte and natural killer cell numbers and functions in healthy young males. Nutrition.

[95] Monk J.M., Hou T.Y., Turk H.F., McMurray D.N., Chapkin R.S. (2013). n3 PUFAs Reduce Mouse CD4(+) T-Cell Ex Vivo Polarization into Th17 Cells. J. Nutr..

[96] Monk J.M., Hou T.Y., Turk H.F., Weeks B., Wu C.D., McMurray D.N., Chapkin R.S. (2012). Dietary n-3 Polyunsaturated Fatty Acids (PUFA) Decrease Obesity-Associated Th17 Cell-Mediated Inflammation during Colitis. PLoS ONE.

[97] Chiurchiu V., Leuti A., Dalli J., Jacobsson A., Battistini L., Maccarrone M., Serhan C.N. (2016). Proresolving lipid mediators resolvin D1, resolvin D2, and maresin 1 are critical in modulating T cell responses. Sci. Transl. Med..

[98] Kim W., Fan Y.Y., Barhoumi R., Smith R., McMurray D.N., Chapkin R.S. (2008). n-3 polyunsaturated fatty acids suppress the localization and activation of signaling proteins at the immunological synapse in murine CD4+ T cells by affecting lipid raft formation. J. Immunol..

[99] Yog R., Barhoumi R., McMurray D.N., Chapkin R.S. (2010). n-3 polyunsaturated fatty acids suppress mitochondrial translocation to the immunologic synapse and modulate calcium signaling in T cells. J. Immunol..

[100] Fan Y.Y., Ly L.H., Barhoumi R., McMurray D.N., Chapkin R.S. (2004). Dietary docosahexaenoic acid suppresses T cell protein kinase C theta lipid raft recruitment and IL-2 production. J. Immunol..

[101] Schieffer D., Naware S., Bakun W., Bamezai A.K. (2014). Lipid raft-based membrane order is important for antigen-specific clonal expansion of CD4(+) T lymphocytes. BMC Immunol..

[102] Hou T.Y., Barhoumi R., Fan Y.Y., Rivera G.M., Hannoush R.N., McMurray D.N., Chapkin R.S. (2016). n-3 polyunsaturated fatty acids suppress CD4(+) T cell proliferation by altering phosphatidylinositol-(4,5)-bisphosphate [PI(4,5)P-2] organization. Biochim. Et. Biophys. Acta-Biomembr..

[103] Fan Y.Y., Fuentes N.R., Hou T.Y., Barhoumi R., Li X.C., Deutz N.E.P., Engelen M.P.K.J., McMurray D.N., Chapkin R.S. (2018). Remodelling of primary human CD4(+) T cell plasma membrane order by n-3 PUFA. Br. J. Nutr..

[104] Jeffery L., Fisk H.L., Calder P.C., Filer A., Raza K., Buckley C.D., McInnes I., Taylor P.C., Fisher B.A. (2017). Plasma Levels of Eicosapentaenoic Acid Are Associated with Anti-TNF Responsiveness in Rheumatoid Arthritis and Inhibit the Etanercept-driven Rise in Th17 Cell Differentiation in Vitro. J. Rheumatol..

[105] Allen M.J., Fan Y.Y., Monk J.M., Hou T.Y., Barhoumi R., McMurray D.N., Chapkin R.S. (2014). n-3 PUFAs Reduce T-Helper 17 Cell Differentiation by Decreasing Responsiveness to Interleukin-6 in Isolated Mouse Splenic CD4(+) T Cells. J. Nutr..

[106] Shoda H., Yanai R., Yoshimura T., Nagai T., Kimura K., Sobrin L., Connor K.M., Sakoda Y., Tamada K., Ikeda T. (2015). Dietary Omega-3 Fatty Acids Suppress Experimental Autoimmune Uveitis in Association with Inhibition of Th1 and Th17 Cell Function. PLoS ONE.

[107] Lian M., Luo W.J., Sui Y.H., Li Z.P., Hua J. (2015). Dietary n-3 PUFA Protects Mice from Con A Induced Liver Injury by Modulating Regulatory T Cells and PPAR-gamma Expression. PLoS ONE.

[108] Woodworth H.L., McCaskey S.J., Duriancik D.M., Clinthorne J.F., Langohr I.M., Gardner E.M., Fenton J.I. (2010). Dietary fish oil alters T lymphocyte cell populations and exacerbates disease in a mouse model of inflammatory colitis. Cancer Res..

[109] Kim J.Y., Lim K., Kim K.H., Kim J.H., Choi J.S., Shim S.C. (2018). N-3 polyunsaturated fatty acids restore Th17 and Treg balance in collagen antibody-induced arthritis. PLoS ONE.

[110] Han S.C., Koo D.H., Kang N.J., Yoon W.J., Kang G.J., Kang H.K., Yoo E.S. (2015). Docosahexaenoic Acid Alleviates Atopic Dermatitis by Generating Tregs and IL-10/TGF-beta-Modified Macrophages via a TGF-beta-Dependent Mechanism. J. Invest. Derm..

[111] Hardy R.R., Hayakawa K. (2001). B cell development pathways. Annu. Rev. Immunol..

[112] Baumgarth N. (2011). The double life of a B-1 cell: Self-reactivity selects for protective effector functions. Nat. Rev. Immunol..

[113] Prieto J.M.B., Felippe M.J.B. (2017). Development, phenotype, and function of non-conventional B cells. Comp. Immunol. Microbiol. Infect. Dis..

[114] Teague H., Fhaner C.J., Harris M., Duriancik D.M., Reid G.E., Shaikh S.R. (2013). n-3 PUFAs enhance the frequency of murine B-cell subsets and restore the impairment of antibody production to a T-independent antigen in obesity. J. Lipid Res..

[115] Teague H., Harris M., Whelan J., Comstock S.S., Fenton J.I., Shaikh S.R. (2016). Short-term consumption of n-3 PUFAs increases murine IL-5 levels, but IL-5 is not the mechanistic link between n-3 fatty acids and changes in B-cell populations. J. Nutr. Biochem..

[116] Tomasdottir V., Thorleifsdottir S., Vikingsson A., Hardardottir I., Freysdottir J. (2014). Dietary omega-3 fatty acids enhance the B1 but not the B2 cell immune response in mice with antigen-induced peritonitis. J. Nutr. Biochem..

[117] Teague H., Harris M., Fenton J., Lallemand P., Shewchuk B.M., Shaikh S.R. (2014). Eicosapentaenoic and docosahexaenoic acid ethyl esters differentially enhance B-cell activity in murine obesity. J. Lipid Res..

[118] Rockett B.D., Harris M., Shaikh S.R. (2012). High dose of an n-3 polyunsaturated fatty acid diet lowers activity of C57BL/6 mice. Prostaglandins Leukot. Essent. Fat. Acids.

[119] Tarlinton D. (2019). B cells still front and centre in immunology. Nat. Rev. Immunol..

[120] Harwood N.E., Batista F.D. (2010). Early events in B cell activation. Annu. Rev. Immunol..

[121] Weise C., Hilt K., Milovanovic M., Ernst D., Ruhl R., Worm M. (2011). Inhibition of IgE production by docosahexaenoic acid is mediated by direct interference with STAT6 and NFkappaB pathway in human B cells. J. Nutr. Biochem..

[122] Ramon S., Gao F., Serhan C.N., Phipps R.P. (2012). Specialized proresolving mediators enhance human B cell differentiation to antibody-secreting cells. J. Immunol..

[123] Rockett B.D., Salameh M., Carraway K., Morrison K., Shaikh S.R. (2010). n-3 PUFA improves fatty acid composition, prevents palmitate-induced apoptosis, and differentially modifies B cell cytokine secretion in vitro and ex vivo. J. Lipid Res..

[124] Rockett B.D., Teague H., Harris M., Melton M., Williams J., Wassall S.R., Shaikh S.R. (2012). Fish oil increases raft size and membrane order of B cells accompanied by differential effects on function. J. Lipid Res..

[125] Shaikh S.R., Edidin M. (2007). Immunosuppressive effects of polyunsaturated fatty acids on antigen presentation by human leukocyte antigen class I molecules. J. Lipid Res..

[126] Verlengia R., Gorjao R., Kanunfre C.C., Bordin S., de Lima T.M., Martins E.F., Newsholme P., Curi R. (2004). Effects of EPA and DHA on proliferation, cytokine production, and gene expression in Raji cells. Lipids.

[127] Gurzell E.A., Teague H., Duriancik D., Clinthorne J., Harris M., Shaikh S.R., Fenton J.I. (2015). Marine fish oils are not equivalent with respect to B-cell membrane organization and activation. J. Nutr. Biochem..

[128] Schraml B.U., Sousa C.R.E. (2015). Defining dendritic cells. Curr. Opin. Immunol..

[129] Wang H., Hao Q., Li Q.R., Yan X.W., Ye S., Li Y.S., Li N., Li J.S. (2007). omega-3 Polyunsaturated fatty acids affect lipopolysaccharide-induced maturation of dendritic cells through mitogen-activated protein kinases p38. Nutrition.

[130] Kong W., Yen J.H., Vassiliou E., Adhikary S., Toscano M.G., Ganea D. (2010). Docosahexaenoic acid prevents dendritic cell maturation and in vitro and in vivo expression of the IL-12 cytokine family. Lipids Health Dis..

[131] Zapata-Gonzalez F., Rueda F., Petriz J., Domingo P., Villarroya F., Diaz-Delfin J., de Madariaga M.A., Domingo J.C. (2008). Human dendritic cell activities are modulated by the omega-3 fatty acid, docosahexaenoic acid, mainly through PPAR(gamma):RXR heterodimers: comparison with other polyunsaturated fatty acids. J. Leukoc. Biol..

[132] Zeyda M., Kirsch B.M., Geyeregger R., Stuhlmeier K.M., Zlabinger G.J., Horl W.H., Saemann M.D., Stulnig T.M. (2005). Inhibition of human dendritic cell maturation and function by the novel immunosuppressant FK778. Transplantation.

[133] Sanderson P., MacPherson G.G., Jenkins C.H., Calder P.C. (1997). Dietary fish oil diminishes the antigen presentation activity of rat dendritic cells. J. Leukoc. Biol..

[134] Zeyda M., Saemann M.D., Stuhlmeier K.M., Mascher D.G., Nowotny P.N., Zlabinger G.J., Waldhausl W., Stulnig T.M. (2005). Polyunsaturated fatty acids block dendritic cell activation and function independently of NF-kappaB activation. J. Biol. Chem..

[135] Wang X.B., Liu J., Wu J.S., Sun Z.M., Huang S.A. (2007). Effects of soluble secreted by acute myeloid leukemia cells on differentiation, maturation, apoptosis, and functions of dendritic cells. Ai. Zheng.

[136] Abel A.M., Yang C., Thakar M.S., Malarkannan S. (2018). Natural Killer Cells: Development, Maturation, and Clinical Utilization. Front. Immunol..

[137] Han L.R., Lei H.N., Tian Z.W., Wang X., Cheng D., Wang C.L. (2018). The immunomodulatory activity and mechanism of docosahexenoic acid (DHA) on immunosuppressive mice models. Food Funct..

[138] Schwerbrock N.M.J., Karlsson E.A., Shi Q., Sheridan P.A., Beck M.A. (2009). Fish Oil-Fed Mice Have Impaired Resistance to Influenza Infection. J. Nutr..

[139] Thies F., Nebe-von-Caron G., Powell J.R., Yaqoob P., Newsholme E.A., Calder P.C. (2001). Dietary supplementation with eicosapentaenoic acid, but not with other long-chain n-3 or n-6 polyunsaturated fatty acids, decreases natural killer cell activity in healthy subjects aged >55 y. Am. J. Clin. Nutr..

[140] Krystel-Whittemore M., Dileepan K.N., Wood J.G. (2015). Mast Cell: A Multi-Functional Master Cell. Front. Immunol..

[141] Latif M.A., Abdul-Hamid M., Galaly S.R. (2015). Effect of diethylcarbamazine citrate and omega-3 fatty acids on trimellitic anhydride-induced rat skin allergy. Asian Pac. J. Allergy Immunol..

[142] Van den Elsen L.W., Nusse Y., Balvers M., Redegeld F.A., Knol E.F., Garssen J., Willemsen L.E. (2013). n-3 Long-chain PUFA reduce allergy-related mediator release by human mast cells in vitro via inhibition of reactive oxygen species. Br. J. Nutr..

[143] Wang X., Ma D.W., Kang J.X., Kulka M. (2015). n-3 Polyunsaturated fatty acids inhibit Fc epsilon receptor I-mediated mast cell activation. J. Nutr. Biochem..

[144] Jang H., Koo J., Park B. (2018). Atopic dermatitis-like skin lesions are suppressed in fat-1 transgenic mice through the inhibition of inflammasomes. Allergy.

[145] Kim T.H., Kim G.D., Jin Y.H., Park Y.S., Park C.S. (2012). Omega-3 fatty acid-derived mediator, Resolvin E1, ameliorates 2,4-dinitrofluorobenzene-induced atopic dermatitis in NC/Nga mice. Int. Immunopharmacol..

[146] Park B.K., Park S., Park J.B., Park M.C., Min T.S., Jin M. (2013). Omega-3 fatty acids suppress Th2-associated cytokine gene expressions and GATA transcription factors in mast cells. J. Nutr. Biochem..

[147] Brannan J.D., Bood J., Alkhabaz A., Balgoma D., Otis J., Delin I., Dahlen B., Wheelock C.E., Nair P., Dahlen S.E. (2015). The Effect of Omega-3 Fatty Acids on Bronchial Hyperresponsiveness, Sputum Eosinophilia, and Mast Cell Mediators in Asthma. Chest.

[148] Yamanishi Y., Miyake K., Iki M., Tsutsui H., Karasuyama H. (2017). Recent advances in understanding basophil-mediated Th2 immune responses. Immunol. Rev..

[149] Jin M., Park S., Park B.K., Choi J.J., Yoon S.J., Yang M., Pyo M.Y. (2014). Eicosapentaenoic Acid and Docosahexaenoic Acid Suppress Th2 Cytokine Expression in RBL-2H3 Basophilic Leukemia Cells. J. Med. Food.

[150] Kawasaki M., Toyoda M., Teshima R., Sawada J., Saito Y. (1994). Effect of Alpha-Linolenic Acid on the Metabolism of Omega-3 and Omega-6 Polyunsaturated Fatty-Acids and Histamine-Release in Rbl-2h3 Cells. Biol. Pharm. Bull..

[151] Cho E., Park Y. (2016). Association between serum fatty acid composition and innate immune markers in healthy adults. Nutr. Res. Pract..

[152] Arm J.P., Boyce J.A., Wang L., Chhay H., Zahid M., Patil V., Govindarajulu U., Ivester P., Weaver K.L., Sergeant S. (2013). Impact of botanical oils on polyunsaturated fatty acid metabolism and leukotriene generation in mild asthmatics. Lipids Health Dis..

[153] Wen T., Rothenberg M.E. (2016). The Regulatory Function of Eosinophils. Microbiol. Spectr..

[154] De Matos O.G., Amaral S.S., da Silva P.E.M.P., Perez D.A., Alvarenga D.M., Ferreira A.V.M., Alvarez-Leite J., Menezes G.B., Cara D.C. (2012). Dietary Supplementation with Omega-3-PUFA-Rich Fish Oil Reduces Signs of Food Allergy in Ovalbumin-Sensitized Mice. Clin. Dev. Immunol..

[155] Mochimaru T., Fukunaga K., Miyata J., Matsusaka M., Masaki K., Kabata H., Ueda S., Suzuki Y., Goto T., Urabe D. (2018). 12-OH-17,18-Epoxyeicosatetraenoic acid alleviates eosinophilic airway inflammation in murine lungs. Allergy.

[156] Yoshida S., Yasutomo K., Watanabe T. (2016). Treatment with DHA/EPA ameliorates atopic dermatitis-like skin disease by blocking LTB4 production. J. Med. Investig..

[157] Hirakata T., Lee H.C., Ohba M., Saeki K., Okuno T., Murakami A., Matsuda A., Yokomizo T. (2019). Dietary omega-3 fatty acids alter the lipid mediator profile and alleviate allergic conjunctivitis without modulating Th2 immune responses. FASEB J..

[158] Moustaka K., Maleskou E., Lambrianidou A., Papadopoulos S., Lekka M.E., Trangas T., Kitsiouli E. (2019). Docosahexaenoic Acid Inhibits Proliferation of EoL-1 Leukemia Cells and Induces Cell Cycle Arrest and Cell Differentiation. Nutrients.

[159] Tanigai T., Ueki S., Kihara J., Kamada R., Yamauchi Y., Sokal A., Takeda M., Ito W., Kayaba H., Adachi T. (2012). Docosahexaenoic Acid Exerts Anti-Inflammatory Action on Human Eosinophils through Peroxisome Proliferator-Activated Receptor-Independent Mechanisms. Int. Arch. Allergy Immunol..

[160] Ostermann A.I., Waindok P., Schmidt M.J., Chiu C.Y., Smyl C., Rohwer N., Weylandt K.H., Schebb N.H. (2017). Modulation of the endogenous omega-3 fatty acid and oxylipin profile in vivo-A comparison of the fat-1 transgenic mouse with C57BL/6 wildtype mice on an omega-3 fatty acid enriched diet. PLoS ONE.

[161] Abbott S.K., Else P.L., Atkins T.A., Hulbert A.J. (2012). Fatty acid composition of membrane bilayers: importance of diet polyunsaturated fat balance. Biochim. Biophys. Acta.

[162] Ferreri C., Masi A., Sansone A., Giacometti G., Larocca A.V., Menounou G., Scanferlato R., Tortorella S., Rota D., Conti M. (2016). Fatty Acids in Membranes as Homeostatic, Metabolic and Nutritional Biomarkers: Recent Advancements in Analytics and Diagnostics. Diagnostics.

